# Factors predicting efficacy of oxaliplatin in combination with 5-fluorouracil (5-FU) ± folinic acid in a compassionate-use cohort of 481 5-FU-resistant advanced colorectal cancer patients

**DOI:** 10.1054/bjoc.2001.1953

**Published:** 2001-08

**Authors:** M A Bensmaïne, M Marty, A de Gramont, S Brienza, F Lévi, M Ducreux, E François, E Gamelin, H Bleiberg, E Cvitkovic

**Affiliations:** Cvitkovic et Associés Consultants, 18–20 rue Pasteur, Kremlin-Bicêtre, 94278; Hôpital Saint-Louis, 1 avenue Claude Vellefaux, Paris, 75010; Hôpital Saint-Antoine, 184 rue du Fg St Antoine, Paris, 75012; Debioclinic, 2 rue du Nouveau Bercy, 94220 Charenton le Pont; Hôpital Paul Brousse, 14 avenue Paul Vaillant Couturier, Villejuif Cedex, 94805; Institut Gustave Roussy, 39 rue Camille Desmoulins, Villejuif Cedex, 94805; Centre Lacassagne, 33 avenue de Valombrose, Nice Cedex 01, 06050; Centre Paul Papin, 2 rue du Moll, Angers Cedex 01, 49033, France and; Institut Jules Bordet, 1 rue Héger-Bordet, Brussels, 1000, Belgium

**Keywords:** clinical resistance, multivariate analysis, salvage chemotherapy

## Abstract

A statistical analysis was performed on the patient data collected from two compassionate-use programmes using oxaliplatin (Eloxatin®) + 5-fluorouracil (5-FU) ± folinic acid (FA), to identify predictive factors for oxaliplatin-based salvage treatment in patients with 5-FU-resistant advanced colorectal cancer (ACRC). 481 5-FU-resistant ACRC patients, most with performance status ≤ 2, ≥ 3 involved sites, and ≥ 2 prior lines of chemotherapy, received oxaliplatin + 5-FU ± FA. Prognostic factors associated with overall response rate (ORR), time to progression (TTP) and overall survival (OS) were identified using univariate and multivariate logistic and/or Cox proportional hazards analyses. The ORR was 16% (95% CI: 13–20), the median TTP was 4.2 months (95% CI: 3.4–4.6), and the median OS was 9.6 months (95% CI: 8.6–10.6). The multivariate analysis indicated poor (≥ 2 WHO) performance status (PS), a large number of prior chemotherapy regimens (≥ 3), a low baseline haemoglobin level (< 10 g/dl), and a triweekly (vs biweekly) treatment administration schedule as significantly associated (*P*< 0.05) with a lower ORR. Sex (male), number of organs involved (≥3) and alkaline phosphatase (AP) level (≥ 2 × the upper limit of normal) were associated (*P*< 0.05) with shorter TTP. Poor PS, a large number of organs involved, and elevated AP were independently and significantly correlated with shorter OS. Our analysis identified a relationship between efficacy results of oxaliplatin + 5-FU ± FA treatment in 5-FU-resistant ACRC patients and baseline prognostic factors related to PS, extent of disease and number of prior regimens. © 2001 Cancer Research Campaign http://www.bjcancer.com


*British Journal of Cancer* (2001) **85**(4), 509–517
© 2001 Cancer Research Campaign
doi: 10.1054/ bjoc.2001.1953, available online at http://www.idealibrary.com on IDEAL

### Factors predicting efficacy of oxaliplatin in combination with 5-fluorouracil (5-FU) ± folinic acid in a compassionate-use cohort of 481 5-FU-resistant advanced colorectal cancer patients

**MA Bensmaïne[1], M Marty[2], A de Gramont[3], S Brienza[4], F Lévi[5], M Ducreux[6], E François[7], E Gamelin[8], H Bleiberg[9] and E Cvitkovic[1,5]**

[1]Cvitkovic et Associés Consultants, 18–20 rue Pasteur, 94278 Kremlin-Bicêtre; [2]Hôpital Saint-Louis, 1 avenue Claude Vellefaux, 75010 Paris; [3]Hôpital Saint-Antoine, 184 rue du Fg St Antoine, 75012 Paris; [4]Debioclinic, 2 rue du Nouveau Bercy, 94220 Charenton le Pont; [5]Hôpital Paul Brousse, 14 avenue Paul Vaillant Couturier, 94805 Villejuif Cedex; [6]Institut Gustave Roussy, 39 rue Camille Desmoulins, 94805 Villejuif Cedex; [7]Centre Lacassagne, 33 avenue de Valombrose, 06050 Nice Cedex 01; [8]Centre Paul Papin, 2 rue du Moll, 49033 Angers Cedex 01, France and [9]Institut Jules Bordet, 1 rue Héger-Bordet, 1000 Brussels, Belgium

**Summary** A statistical analysis was performed on the patient data collected from two compassionate-use programmes using oxaliplatin (Eloxatin®) + 5-fluorouracil (5-FU) ± folinic acid (FA), to identify predictive factors for oxaliplatin-based salvage treatment in patients with 5-FU-resistant advanced colorectal cancer (ACRC). 481 5-FU-resistant ACRC patients, most with performance status ≤ 2, ≥ 3 involved sites, and ≥ 2 prior lines of chemotherapy, received oxaliplatin + 5-FU ± FA. Prognostic factors associated with overall response rate (ORR), time to progression (TTP) and overall survival (OS) were identified using univariate and multivariate logistic and/or Cox proportional hazards analyses. The ORR was 16% (95% CI: 13–20), the median TTP was 4.2 months (95% CI: 3.4–4.6), and the median OS was 9.6 months (95% CI: 8.6–10.6). The multivariate analysis indicated poor (≥ 2 WHO) performance status (PS), a large number of prior chemotherapy regimens (≥ 3), a low baseline haemoglobin level (< 10 g/dl), and a triweekly (vs biweekly) treatment administration schedule as significantly associated ($P < 0.05$) with a lower ORR. Sex (male), number of organs involved (≥3) and alkaline phosphatase (AP) level (≥ 2 × the upper limit of normal) were associated ($P < 0.05$) with shorter TTP. Poor PS, a large number of organs involved, and elevated AP were independently and significantly correlated with shorter OS. Our analysis identified a relationship between efficacy results of oxaliplatin + 5-FU ± FA treatment in 5-FU-resistant ACRC patients and baseline prognostic factors related to PS, extent of disease and number of prior regimens. © 2001 Cancer Research Campaign http://www.bjcancer.com

**Keywords**: clinical resistance; multivariate analysis; salvage chemotherapy

The prognosis for colorectal cancer (CRC) is generally poor, and although surgery is an effective treatment, 50% of patients either have metastatic or inoperable disease at the initial diagnosis or else develop metastases and/or local recurrent disease within several months (Boring et al, 1992). Although 5-fluorouracil (5-FU) is a moderately effective chemotherapeutic option, many patients either do not respond or progress after a brief response, and therefore an effective second-line chemotherapy option is needed.

Until 1990, the search for salvage therapies for CRC patients failing 5-FU-based chemotherapy regimens focused on new 5-FU administration modalities. Continuous infusion and high-dose (HD) 5-FU were investigated, but showed no significant benefit in terms of efficacy, and its modulation using folinic acid (FA), although showing an improved response rate, showed no benefits in terms of survival (Advanced Colorectal Cancer Meta-Analysis Project, 1992). The combination of 5-FU with other cytotoxic agents, such as cisplatin, or interferon was also explored, with no improvement in efficacy, and increased toxicity and morbidity (Whitehead et al, 1991; Behrens et al, 1989; LoRusso et al, 1989; Petrelli et al, 1989; Iyer et al, 1988; Hill et al, 1995). Since 1990, the introduction of several new classes of antineoplastic agents active in CRC patients, including oxaliplatin and irinotecan, has opened up new therapeutic possibilities.

Oxaliplatin – trans-*l*-diaminocyclohexane (DACH) oxalatoplatinum (Eloxatin®, Sanofi-Synthelabo, France) – is a DACH platinum derivative whose main mechanism of action is the formation of DNA intrastrand adducts between two adjacent guanines d(GpG) or adjacent guanine and adenine d(GpA) bases.

In vitro and in vivo data have shown oxaliplatin to be active against CRC cell lines and synergistic with 5-FU, even in 5-FU resistant cell lines (Raymond et al, 1998a; Raymond et al, 1998b). Like other DACH platinums, it has also been shown to be only partially cross-resistant with cisplatin or carboplatin in various preclinical studies. Oxaliplatin is the first platinum compound to show activity as a single agent when used in the treatment of advanced CRC patients (Lévi et al, 1993). In two phase II studies involving patients with fluoropyrimidine-resistant advanced CRC,

*Received 8 December 2000*
*Revised 17 May 2001*
*Accepted 1 June 2001*

*Correspondence to:* E Cvitkovic

Results from this study have been partially presented at the Annual Meeting of the American Society of Clinical Oncology, Atlanta, May 1999, the 8th International Symposium on Platinum and other Metal Coordination Compounds in Cancer Chemotherapy, Oxford, March 1999, and at the Annual Meeting of the American Society of Clinical Oncology, May 1996.

treated with oxaliplatin given as a single agent at 130 mg/m$^2$ over a 2-h infusion every 3 weeks, the objective response rates (ORR) were both 10%, with 24% and 40% of patients exhibiting disease stabilization, and median overall survival times (OS) of 8.3 and 10 months (Machover et al, 1996). In a phase II trial by Bécouarn et al, involving untreated CRC patients, oxaliplatin used as a single agent produced a 27% ORR (95% CI: 13.8–44.1%), with a median response duration of 195 days (range 126–364+), median time to progression (TTP) of 127 days (range 22–364+), and median OS of 395 days (range: 28–573+) (Becouarn et al, 1998).

Oxaliplatin has also been given to patients progressing on 5-FU-based regimens in phase II trials. Its addition to 5-FU + FA treatment has produced ORRs ranging from 11% to 58%, depending on patient characteristics and treatment scheme, with a median progression-free survival (PFS) ranging from 7 to 10 months, and a median OS of 12 to 17 months (De Gramont et al, 1997; Gerard et al, 1997; Meyer et al, 1997; Levi et al, 1992; Bertheault-Cvitkovic et al, 1996; Garufi et al, 1995; André et al, 1999).

In a randomized phase III trial reported by Giacchetti et al, 200 non-pretreated metastatic CRC patients were given a chronomodulated 5-FU + FA regimen either with or without the addition of oxaliplatin at 125 mg/m$^2$ q 3 weeks. The ORR was 53% in the oxaliplatin arm (95% CI: 42–63%), and 16% in the oxaliplatin-free arm (95% CI: 9–24%; $P < 0.001$). The median PFS in the oxaliplatin and control arms were 7.9 and 4.3 months, respectively, and the OS was 17.6 and 19.4 months, respectively (Giacchetti et al, 2000). In another phase III randomized study, reported by de Gramont et al, involving 420 previously untreated metastatic CRC patients, the ORR was found to be 51% in the oxaliplatin-containing arm and 23% in the control arm, with the median PFS being 8.7 months in the oxaliplatin arm and 6.1 months in the other, and with an OS of 15.9 and 14.7 months respectively (De Gramont et al, 2000). While a survival advantage in first-line therapy has not yet been demonstrated for oxaliplatin, as is contrariwise the case for irinotecan (Douillard et al, 2000; Saltz et al, 2000), investigation of oxaliplatin-based combinations in pretreated and untreated CRC patients continues, and the present article reports the activity of oxaliplatin + 5-FU ± FA in 5-FU resistant CRC patients treated in the realistic clinical practice setting of compassionate-use programmes.

The compassionate-use programmes which are the subject of this article comprised two cohorts, totalling 696 patients whose disease had progressed on prior 5-FU-based chemotherapy. The first European-wide cohort of 206 patients received oxaliplatin between January 1994 and June 1995. Oncologists who wanted to administer oxaliplatin to specific patients under this programme applied to the programme's sponsor. The second cohort of 490 patients were treated with oxaliplatin in France under an *Autorisation Temporaire d'Utilisation* (ATU) starting in September 1995 and implemented during the French application review period, and continuing until marketing approval for the new drug was granted in France (April 1996). All requests were monitored by the French *Agence du médicament*. The two patient cohorts of 5-FU-resistant patients treated with oxaliplatin plus 5-FU ± FA, which had already been the subject of safety assessments in pursuit of their primary regulatory objective, were analysed as a single group in order to identify factors predicting the efficacy of oxaliplatin when added to 5-FU ± FA in a real-life prescription context, including patients with source-reviewed and third-party-assessed evidence of clinical 5-FU resistance. Efficacy has been analysed in terms of ORR, median TTP and OS.

### MATERIALS AND METHODS

### Compassionate-use programme participants

A total of 696 advanced CRC patients were recruited in both compassionate-use programmes. All the patients received oxaliplatin with 5-FU ± FA. The first patient cohort, consisting of 206 patients, was treated in 44 European centres. The second patient cohort, consisting of 490 patients was treated in 148 French institutions.

### Diagnosis and main inclusion guidelines

The main inclusion criterion for the ATU was histologically proven advanced/metastatic CRC. Patients were also to have objective, verifiable resistance to 5-FU ± FA (on-treatment progression) or to have contraindications for treatment with 5-FU, in which case they were given oxaliplatin as a single agent. There were no restrictions on age, performance status, or the number of prior chemotherapy regimens.

### Data collection and patient classification

All cases in the present cohort were thoroughly reviewed on-site by a team of medical oncologists and all data were source-verified.

Table 1 summarizes the information requested for each patient in order for the physician and sponsor to identify which patients were to receive oxaliplatin. After source review, all case report forms were independently verified for completeness and consistency. For each patient, the team reviewed prior therapies, evidence of resistance to prior 5-FU treatment, dose and schedule of 5-FU ± FA before and during oxaliplatin treatment, as well as tumour response to the oxaliplatin-based treatment.

In addition, an external panel of radiologists verified available imaging evidence of progression during prior 5-FU-based treatment for all patients included in the present cohort. Furthermore, they reviewed all the oxaliplatin-treated patients whose disease was reported to be responding or who showed stabilisation (≥ 9 weeks or 3 planned cycles).

### Identification of 5-FU-resistance

After verification of patients' characteristics, disease history and disease progression under prior 5-FU treatment, and after the

**Table 1** Information collected for each patient

| |
|---|
| Patient identification |
| Start date of oxaliplatin-based treatment |
| Age/sex |
| Performance status |
| Primary tumour site (colon or rectum) |
| Prior therapies, especially chemotherapy |
| Number of prior chemotherapy regimens |
| Dose/schedule details of prior 5-FU-based treatment |
| Organs involved |
| Proof of progression while on prior 5-FU-based treatment |
| Dose/schedule of Oxaliplatin +5-FU ± FA treatment |
| Safety of oxaliplatin treatment |
| Overall response while on oxaliplatin treatment |
| Progression, with proof, if applicable |
| Survival status at last visit |
| Cause of death, if applicable |

 *© 2001 Cancer Research Campaign*

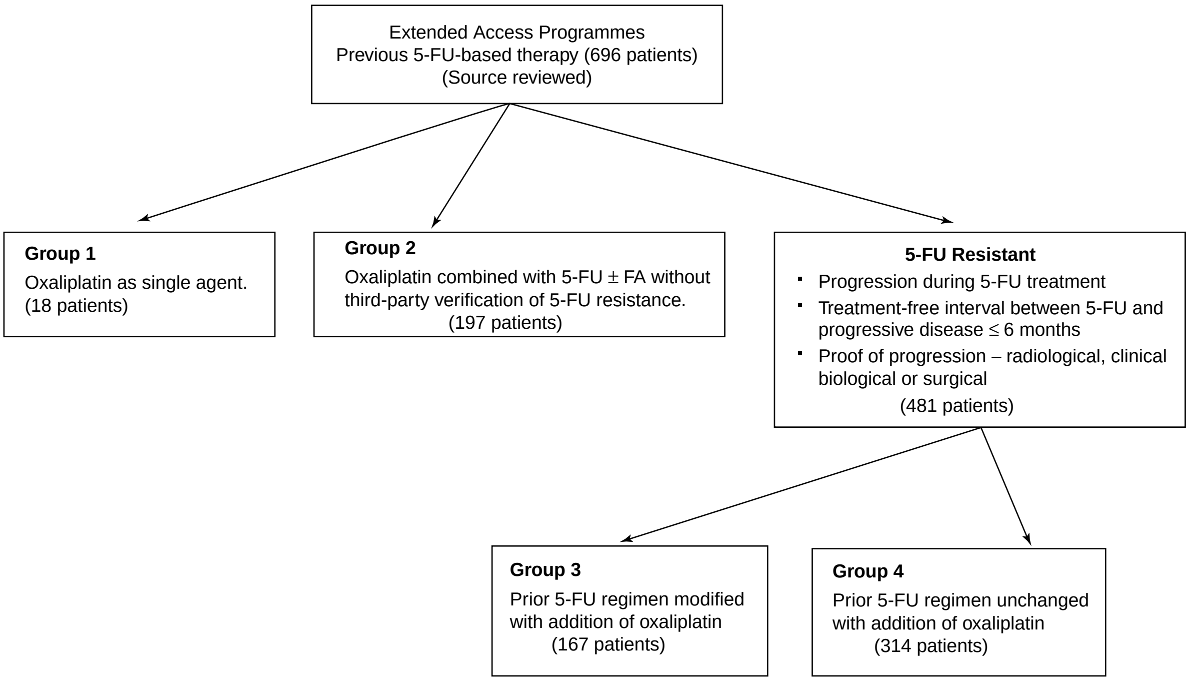

**Figure 1** Criteria used to identify patient resistance to 5-FU

external panel review, an algorithm was applied, based on treatment modality and clinical, radiological evidence of 5-FU-resistance, to reliably identify patients with 5-FU-resistant disease (Figure 1). As a result of this assessment, 481 5-FU-resistant advanced CRC patients were identified. These patients are the subject of the univariate and multivariate analyses presented in this article.

### Classification of 5-FU-based chemotherapy regimens

Administration and schedule modalities for 5-FU (and for FA when used as a modulating agent) were systematically recorded for every previous regimen. 5-FU was administered according to the preference of the individual prescribers, and hence in a very wide variety of doses and schedules, both as a single agent and in combination with various other cytotoxic or modulating agents. For the purposes of the present analysis, the administration of 5-FU (with or without FA), was defined according to the schedules encountered in the patient cohort, as follows:

- Bolus/short infusion administration (≤ 4 h) ± FA: either daily (2–5 consecutive days), repeated weekly or every 3–4 weeks.
- High-dose-intensity intermittent infusional schedule: this encompassed all intravenous infusions ≥ 8 h and ≤ 48 h, given at weekly or biweekly intervals, usually involving high total doses of 5-FU (≥ 1000 mg/m$^2$), ± FA. They were subdivided into: 8-h weekly infusion, 48-h biweekly infusion (± 5-FU bolus), and 24-h infusion, given weekly.
- Continuous infusion: Intravenous continuous infusion (< 1 000 mg/m$^2$), ± FA for at least 4 days. These comprised: 4/5 days every 2–3 weeks given at a flat rate of delivery, 4/5 days continuous chronomodulated delivery, given every 2–3 weeks, + FA, or protracted continuous infusion lasting for more than 10 consecutive days ± FA.

### Oxaliplatin dosing

Oxaliplatin was administered either biweekly at doses ranging from 80 to 100 mg/m$^2$/cycle, usually associated with high-dose infusional intermittent schedules, or every 3–4 weeks at doses ranging from 100 to 130 mg/m$^2$/cycle, associated with a wide variety of 5-FU schedules and administration modalities. Prescribers chose the schedule of oxaliplatin, all with similar planned dose intensities (33–42 mg/m$^2$/week), that they considered best adapted to the associated 5-FU-based regimen.

### Efficacy criteria

Tumour response was assessed in conformity with World Health Organization (WHO) criteria (Miller et al, 1981). Nevertheless, as this was an ATU in unselected, heavily pretreated patients, the assessments of antitumoural efficacy were not implemented at regular intervals as in a formal clinical trial. The responses were therefore defined according to the following modified WHO criteria (modifications in italics):

- Complete Response (CR): complete regression of all evidence of tumour, where possible assessed in two evaluations at least 1 month apart.
- Partial Response (PR): a ≥ 50% decrease in the sum of the products of the two longest perpendicular diameters of all measurable lesions with no increase in size of tumour or appearance of any new lesion. *As long as patients showed no clinical or biological signs of progression in the 8 weeks following their determination of PR, they were maintained in this category*.
- Stable Disease (SD): < 50% decrease of measurable disease and ≤ 25% progression of measurable lesion, *as well as no clinical, biological or radiological evidence of any new lesion*.

© 2001 Cancer Research Campaign

- Progressive Disease (PD): ≥ 25% increase in the sum of the products of two diameters of one or more measurable lesions, evidence of new lesions, *death from disease progression within 8 weeks of introduction of oxaliplatin or lack of formal disease evaluation after 3 cycles of treatment*.

SD is a clinically relevant category in this analysis, as most patients treated under this compassionate-use programme exhibited PD under their previous treatment regimen. SD represents a positive change in disease status for such patients, who have a similar median OS to those exhibiting PR (Graf et al, 1994).

Time-related parameters were also calculated, with median TTP and OS being defined as the time elapsed between the start of oxaliplatin-based treatment and the date when evidence of tumour progression (clinical and/or radiological) was first obtained and the date of patient's death, respectively.

### Methods for statistical analysis

TTP and OS curves for the various groups were computed using the Kaplan-Meier method. Both univariate and multivariate analyses were used to assess potential prognostic factors. For univariate analyses, dichotomous (objective responses) and censored (TTP and OS) data were compared by means of Pearson's $\chi^2$ test and the log-rank test, respectively. The variables included in the univariate analysis were: sex, age (< 60 or ≥ 60 years), PS, primary tumour site (colon or rectum), number of involved organs (1, 2, or ≥ 3), number of prior chemotherapy regimens for CRC (1, 2, ≥ 3), haemoglobin level, (< 10 g/dl or ≥ 10 g/dl), serum alkaline phosphatase (AP) level (< 2 × ULN or ≥ 2 x ULN), oxaliplatin schedule (biweekly or triweekly), change of 5-FU scheme (yes or no), type of 5-FU + oxaliplatin delivery, (bolus, HD or continuous infusion), study treatment regimen (oxaliplatin + 5-FU, or oxaliplatin + 5-FU + FA), and patient cohort (European or French). For the multivariate analysis, dichotomous and censored data were compared by means of a multivariate logistic model (Cox, 1970) and a Cox proportional hazards model (Cox, 1972), respectively. A forward stepwise procedure was used, based on the Wald statistic. The significance level used as a criterion for retaining variables from the univariate analysis for inclusion in the multivariate analysis was $P \leq 0.10$.

### RESULTS

### Cohort characteristics

Out of the 696 patients recruited, 481 (69%) were considered, after source-data review and third-party verification, as having 5-FU-resistant advanced CRC; 111 in the initial European ATU cohort and 370 in the second French ATU cohort. Out of the 481 patients confirmed as 5-FU resistant, 443 (92%) of these judgements were based on radiological evidence of progression. The overall group was 60% male, and 56% were over 60 years old. Only 9 of the patients (2%) had a PS > 2 and 53 (11%) had a PS = 2 (see Table 2 for details of the patients' characteristics).

**Table 2** Patient pretreatment characteristics

| Characteristic | European cohort *n* = 111 | French cohort *n* = 370 | All patients *n* = 481 |
|---|---|---|---|
| Sex | | | |
| Men/Women | 58 / 53 | 230 / 140 | 288 / 193 |
| Age | | | |
| < 60 / ≥60 years | 53 / 58 | 159 / 211 | 212 / 269 |
| WHO PS | | | |
| 0 / 1 / 2 / 3 / MD | 46 / 46 / 14 / 5 / 0 | 144 / 168 / 39 / 4 / 15 | 190 / 214 / 53 / 9 / 15 |
| Primary tumour site | | | |
| Colon/rectum | 69/42 | 259 / 111 | 328 / 153 |
| Initial Duke's stage | | | |
| A / B / C / D / MD | 2 / 11 / 26 / 70 / 2 | 3 / 48 / 118 / 193 / 8 | 5 / 59 / 144 / 263 / 10 |
| Number of metastatic sites | | | |
| 1 / 2 / ≥3 / MD | 48 / 31 / 32 / 0 | 188 / 127 / 51 / 4 | 236 / 158 / 83 / 4 |
| Organs involved | | | |
| Liver | 93 | 292 | 385 |
| Lung | 43 | 112 | 155 |
| Lymph nodes | 31 | 51 | 82 |
| Peritoneum | 14 | 56 | 70 |
| Other | 14 | 24 | 38 |
| Number of prior CT lines | | | |
| 1 / 2 / ≥3 | 59 / 26 / 26 | 123 / 132 / 115 | 182 / 158 / 141 |
| Patients receiving 1 prior CT line in adjuvant setting as only prior treatment | 9 | 14 | 23 |
| Interval between diagnosis of recurrence and oxaliplatin treatment | | | |
| 0–≤ 12 months | 59 | 182 | 241 |
| >12–≤ 24 months | 37 | 107 | 144 |
| >24 months | 15 | 81 | 96 |
| Interval between last CT cycle and oxaliplatin treatment | | | |
| 0–≤ 3 months | 95 | 291 | 386 |
| > 3–≤ 6 months | 14 | 67 | 81 |
| > 6 months | 2 | 12 | 14 |

MD, missing data; CT, chemotherapy.

*© 2001 Cancer Research Campaign*

**Table 3** Univariate analysis of objective response rate (ORR), time to progression (TTP), and overall survival (OS) following the addition of oxaliplatin to a 5-FU ± FA regimen in 5-FU-resistant colorectal cancer patients

| Parameter | Number of patients | ORR | | TTP | | OS | |
|---|---|---|---|---|---|---|---|
| | | % | *P* | Median (mo) | *P* | Median (mo) | *P* |
| All patients | 481 | 16.4 | | 4.2 | | 9.6 | |
| Cohort | | | | | NS | | NS |
| Europe (first) | 111 | 22.5 | 0.048 | 4.1 | | 9.6 | |
| France (second) | 370 | 14.6 | | 4.3 | | 9.7 | |
| Sex | | | NS | | | | NS |
| Men | 288 | 16.7 | | 4.1 | 0.0305 | 9.5 | |
| Women | 193 | 16.1 | | 4.8 | | 9.9 | |
| Age | | | NS | | NS | | NS |
| <60 | 212 | 16.0 | | 4.3 | | 10.2 | |
| ≥60 | 269 | 16.7 | | 4.2 | | 9.3 | |
| Performance status[a] | | | | | | | |
| 0 | 190 | 21.1 | 0.024 | 4.6 | <0.0001 | 12.5 | <0.0001 |
| 1 | 214 | 13.6 | | 4.3 | | 9.3 | |
| 2 | 53 | 9.4 | | 2.8 | | 4.6 | |
| 3 | 9 | 0 | | 0.8 | | 1 | |
| 0–1 | 404 | 17.1 | 0.071 | 4.5 | <0.0001 | 10.6 | <0.0001 |
| 2–3 | 62 | 8.1 | | 1.9 | | 3.6 | |
| 0 | 190 | 21.1 | 0.013 | 4.6 | <0.0001 | 12.5 | <0.0001 |
| 1–2 | 267 | 12.7 | | 4.0 | | 8.6 | |
| 3 | 9 | 0 | | 0.8 | | 1.0 | |
| Primary tumour site | | | NS | | NS | | NS |
| Colon | 328 | 15.9 | | 4.1 | | 9.6 | |
| Rectum | 153 | 17.6 | | 4.5 | | 9.8 | |
| Number of disease sites[a] | | | NS | | | | |
| 1 | 236 | 16.5 | | 4.7 | <0.0001 | 11.9 | <0.0001 |
| 2 | 158 | 15.8 | | 4.2 | | 9.6 | |
| ≥3 | 83 | 16.9 | | 2.8 | | 6.2 | |
| 1 | 236 | 16.5 | NS | 4.7 | 0.0001 | 11.9 | 0.0004 |
| ≥2 | 241 | 16.2 | | 3.8 | | 8.2 | |
| Number of prior CT lines | | | | | NS | | |
| 1 | 182 | 23.6 | <0.0001 | 4.6 | 0.0309 | 10.7 | |
| 2 | 158 | 16.5 | | 4.6 | | 9.1 | |
| ≥3 | 141 | 7.1 | | 3.2 | | 9.8 | |
| Haemoglobin | | | | | | | |
| <10 g/dl | 50 | 4.0 | 0.012 | 2.3 | 0.0584 | 6.7 | 0.0123 |
| ≥10 g/dl | 416 | 18.0 | | 4.5 | | 9.9 | |
| Serum AP[a] | | | | | | | |
| <2 × ULN | 308 | 19.5 | 0.006 | 4.5 | 0.0175 | 11.0 | <0.0001 |
| ≥ 2 × ULN | 119 | 8.4 | | 3.8 | | 7.4 | |
| Oxaliplatin schedule | | | | | NS | | NS |
| Biweekly | 85 | 29.4 | 0.001 | 5.4 | | 11.1 | |
| Triweekly | 370 | 14.6 | | 4.1 | | 9.9 | |
| Change of 5-FU scheme | | | NS | | NS | | NS |
| Yes | 167 | 15.0 | | 4.4 | | 9.9 | |
| No | 314 | 17.2 | | 4.1 | | 9.6 | |
| 5-FU delivery + oxaliplatin[a] | | | NS | | NS | | NS |
| Bolus | 77 | 13.0 | | 4.3 | | 9.1 | |
| High dose | 273 | 15.8 | | 3.9 | | 9.3 | |
| Continuous infusion | 58 | 17.2 | | 4.7 | | 10.4 | |
| Oxaliplatin[a] | | | | | NS | | NS |
| +5-FU | 374 | 14.7 | 0.070 | 4.2 | | 9.9 | |
| +5-FU +FA | 104 | 22.1 | | 4.1 | | 9.5 | |

[a]Missing data. NS, not significant; CI, confidence interval; AP, alkaline phosphatase; CT, chemotherapy; mo, months; ULN, Upper Limit of Normal.

Out of the 471 patients for whom data concerning staging at diagnosis was available, 64 (14%) initially presented with a Duke's stage of A or B, 144 (31%) with stage C, and 263 (56%) with stage D. Apart from the 4 patients for whom data on metastases were missing, 236 (49%) had only one metastatic site, 158 (33%) had two, and 83 (17%) had three or more. Involvement of the liver predominated, being found in 385 patients (80%), followed by the lung in 155 patients (32%), lymph nodes in 82 patients (17%) and the peritoneum in 70 patients (15%).

There were 141 patients (29%) who had received 3 or more lines of chemotherapy prior to treatment with oxaliplatin, 158 (33%) who had received 2, and 182 patients (38%) who had received 1 prior chemotherapy regimen. Adjuvant chemotherapy was administered to 140 patients (29%). The time interval between the last

© 2001 Cancer Research Campaign

**Table 4** Multivariate analysis of objective response rate (ORR), time to progression (TTP), and overall survival (OS) following the addition of oxaliplatin to a 5-FU ± FA regimen in 5-FU-resistant colorectal cancer patients

| | ORR (*n* = 393) | | TTP (*n* = 408) | | OS (*n* = 412) | |
|---|---|---|---|---|---|---|
| **Parameter** | ***P*** | **Relative risk [95% CI]** | ***P*** | **Relative risk [95% CI]** | ***P*** | **Relative risk [95% CI]** |
| Cohort | NS | | NA | | NA | |
| Europe (first) | | | | | | |
| France (second) | | | | | | |
| Sex | NA | | 0.0022 | 0.71[0.58–0.89] | NA | |
| Male | | | | | | |
| Female | | | | | | |
| PS | 0.005 | 3.01 [1.62–5.60] | 0.0002 | 1.75 [1.30–2.36] | <0.0001 | 2.34 [1.68–3.24] |
| 0–1 | | | | | | |
| 2–3 | | | | | | |
| Number of disease sites | NA | | 0.0005 | | <0.0001 | |
| 1 | | | – | – | – | |
| 2 | | | 0.0409 | 1.27 [1.01–1.60] | 0.0909 | 1.27 [0.96–1.67] |
| ≥3 | | | 0.0001 | 1.75 [1.31–2.33] | <0.0001 | 2.05 [1.50–2.80] |
| Number of prior lines of chemotherapy | 0.0134 | | NS | | NA | |
| 1 | – | | | | | |
| 2 | NS | | | | | |
| ≥3 | 0.0054 | 0.39 [0.20–0.76] | | | | |
| Haemoglobin | 0.0018 | 0.37 [0.20–0.69] | NS | | NS | |
| <10 g/dl | | | | | | |
| ≥10 g/dl | | | | | | |
| Serum alkaline phosphatase | NS | | 0.067 | 1.37 [1.09–1.72] | <0.0001 | 1.88 [1.45–2.44] |
| <2 × ULN | | | | | | |
| ≥2 × ULN | | | | | | |
| Oxaliplatin | NS | | NA | | NA | |
| +5-FU | | | | | | |
| +5-FU + FA | | | | | | |
| Rhythm | <0.0001 | 0.18 [0.10–0.30] | NA | | NA | |
| Biweekly | | | | | | |
| Triweekly | | | | | | |

NS, not significant; NA, not analysed; CI, confidence interval; ULN, Upper Limit of Normal.

regimen and the beginning of oxaliplatin treatment was less than three months for 386 of the patients (80%), between 3 and 6 months for 81 (17%) and greater than 6 months for the remaining 14 patients (3%).

### Antitumour activity

In the European cohort of 111 5-FU-resistant patients, an objective response to oxaliplatin with 5-FU with or without FA was reported in 25 patients, giving a 23% ORR (95% CI: 15–31%). For the French cohort of 370 patients, an objective response to oxaliplatin-based treatment was reported in 54 patients, resulting in a 15% ORR. (95% CI:11–18%). The ORR reported for both groups combined (481 patients) was 16% (95% CI:14–20%). Response rates and time-related parameters according to various variables analysed are presented in Table 3, and the results of the multivariate analysis are presented in Table 4.

Disease stabilization (SD) was recorded in 185 patients of the combined cohort of 481 (38%), of whom 161 (87%) had a TTP of more than 16 weeks. The median duration of SD was 5.7 months (range, 1.5–25.8).

According to the univariate analysis, having a PS of 0 was found to be positively correlated with ORR ($P = 0.024$), and according to the multivariate analysis, having a PS of 0–1 was highly significant ($P = 5 \times 10^{-4}$). The number of prior chemotherapy regimens received was negatively correlated with ORR achieving high significance in the univariate analysis ($P < 1 \times 10^{-4}$). It is of note that an ORR of 23.6% was recorded in patients having received one prior chemotherapy regimen, while according to the multivariate analysis, receiving ≥ 3 prior chemotherapy regimens was highly correlated with a lower likelihood of response (ORR = 7% vs 24% for 1 regimen, $P = 0.0054$).

Haemoglobin level < 10 g/dl at baseline showed a significant negative correlation with ORR (4% vs 18% for ≥ 10 g/dl) in both the univariate ($P = 0.012$) and multivariate ($P = 0.0018$) analyses. Although a baseline serum AP level < 2 × the upper limit of normal (ULN) was highly positively correlated with ORR (20% vs 8% for ≥ 2 ×ULN) according to the univariate analysis, it had no independent significance in the multivariate analysis.

Biweekly oxaliplatin + 5-FU ±FA schedules were found to be positively correlated with ORR (29% vs 15% for the triweekly schedules) and was identified as a highly significant factor for the likelihood of objective response in both analyses. Oxaliplatin administered with both 5-FU and FA, compared to oxaliplatin with 5-FU alone (mostly as a continuous infusion, either 4–5 days q 3 weeks or a protracted continuous IV) exhibited a trend towards higher ORR (22% vs 15%, respectively), but this relation failed to reach statistical significance.

### Time-related parameters

Female sex was found to be positively and significantly correlated with TTP (4.8 months vs 4.1 for male patients) in both the

© 2001 Cancer Research Campaign univariate and the multivariate analysis. Having a PS of 0 was positively correlated with both TTP and OS: in the univariate analysis, PS = 0 was found to be a highly significant factor ($P < 1 \times 10^{-4}$) for both TTP (median 4.6 months) and OS (median 12.4 months), and the multivariate analysis confirmed its independent prognostic value. The number of involved organs had significant prognostic correlations, with single organ involvement being positively correlated with both median TTP and OS in the univariate and multivariate analyses, when compared to patients with either 2 or ≥ 3 involved organs

Although having received only one prior chemotherapy regimen was positively correlated with median TTP (4.6 months), when compared to patients receiving ≥ 2 regimens (3.2 months) by univariate analysis ($P = 0.031$), this factor lost its significance in the multivariate analysis. The number of prior chemotherapy regimens failed to show statistical significance for OS in either analysis. Baseline haemoglobin <10 g/dl was negatively correlated with TTP (median 2.3 months vs 4.5 for ≥ 10 g/dl) and significantly so for OS (median 6.7 months *v* 9.9 for ≥ 10 g/dl), but the multivariate analysis did not show this factor to be significant. Serum AP level < 2 × ULN was significantly positively correlated with TTP (median 4.5 months vs 3.8 for ≥ 2 × ULN) and the correlation was highly significant for OS (median 11.0 months vs 7.4 for ≥2 × ULN; $P < 1 \times 10^{-4}$). The multivariate analysis confirmed its independent prognostic value.

### DISCUSSION

The aim of the present statistical analysis was to identify factors that will serve as prognostic indicators for oxaliplatin/5-FU patients who have proven resistant to 5-FU. Once the first-line 5-FU regimen had failed, the only alternatives with some evidence of activity available at the time were modulations of the 5-FU regimen through addition of FA or interferon, or the administration of the 5-FU-containing treatment using different doses and/or schedules (Streit et al, 1997; Izzo et al, 1994; Falcone et al, 1994; Iyer et al, 1988). At the time of the first cohort's treatment, irinotecan had not yet been approved for this indication, and so the therapeutic choices for CRC patients failing 5-FU-based treatment were extremely limited. Thus, only 3 patients in the European cohort (2.7%) had previously received irinotecan, while 67 patients (18.1%) of the subsequent French cohort had done so, reflecting the timing of compassionate-use and commercial availability of irinotecan.

We should stress that 62% of the patients had already received two or more lines of chemotherapy for their advanced CRC prior to the administration of the oxaliplatin-containing treatment. Furthermore, the number of prior lines of chemotherapy was correlated with another possibly relevant variable; the interval between the diagnosis of metastatic disease and the introduction of the oxaliplatin treatment. The univariate analysis revealed a significant difference in ORR ($P < 0.05$) between the 2 cohorts (23% for the European cohort, 15% for the French). This result is probably associated with the difference in the median number of lines of prior chemotherapy between the two groups (1 for the European cohort, and 2 for the French).

The irinotecan pretreated patients of the French cohort registered an ORR of only 6.0% versus 20.0% in irinotecan-naive patients. However, the higher rate of exposure to irinotecan in the second cohort would not appear to explain the difference for ORR between the two cohorts, as irinotecan pretreatment was not found to be an independent prognostic factor for ORR in a multivariate analysis carried out on the French cohort (data not shown). Nevertheless, irinotecan pretreatment was a predictor of shorter TTP and OS in that cohort. Median TTP in irinotecan pretreated and non-pretreated patients of the French cohort was 2.4 and 4.5 months, respectively, and median OS 6.6 and 9.0 months. Given the arrival of irinotecan as a standard agent in first-line treatment of metastatic CRC, with significant consequences for therapeutic options in progressing patients, the optimization of sequential therapeutic possibilities is a pressing need, and is the subject of an ongoing phase III comparison of irinotecan + 5-FU/FA followed by oxaliplatin + 5-FU/FA versus the inverse sequence (Tournigand et al, 2001).

When considering baseline patient and disease characteristics, PS is clearly a major predictive factor for response, TTP and OS in 5-FU-resistant CRC patients. The number of prior chemotherapy regimens is a key factor for likelihood of response, but is less indicative of the duration of TTP and OS. Haemoglobin level at baseline was significantly linked to response rate, although the significance of this factor for TTP and OS elicited by the univariate analysis was not confirmed by the multivariate assessment. Similarly, although serum AP level proved to be a significant factor for response rate according to the univariate analysis, this was not confirmed by the multivariate analysis. Nevertheless, this variable is independently correlated with both TTP and, more importantly, OS and is thus clinically relevant.

As far as administration regimens and schedules are concerned, the multivariate analysis suggested that biweekly oxaliplatin + FU ± FA administration schedules were a highly significant positive factor for obtaining a response. However, this factor was not significant as far as TTP and OS were concerned. Since there were no guidelines for the frequency of response assessments, and since there is a tendency to higher frequency assessments in the centres that enrol the greatest numbers of patients, the clinical relevance of this statistical relationship remains moot. For example, the French hospitals Saint Antoine and Paul Brousse, being the institutions with the greatest experience with oxaliplatin + 5-FU ± FA, treated a large number of patients in this compassionate-use programme due to the absence of clinical trials at the time, or patient ineligibility for available trials. These patients were nevertheless managed in the same manner as those included in the formal clinical trials. Likewise, the analysis revealed a tendency towards a higher ORR in biweekly regimens, but there is no prospectively controlled data available to confirm this result. Interestingly, changing the 5-FU regimen when oxaliplatin was introduced into the treatment had no bearing on ORR, TTP or OS, calling into question the value of any specific strategy that might be pursued when associating oxaliplatin with a prior 5-FU regimen.

When the data from this study are compared with the efficacy data obtained in formal phase II studies in 5-FU-pretreated CRC patients, which necessarily enforce positive selection through restrictive eligibility criteria, the time-related efficacy parameters are similar, but the ORR is lower (16.4% vs 24–55%) (De Gramont et al, 1997; Gerard et al, 1997; Meyer et al, 1997; Levi et al, 1992; Bertheault-Cvitkovic et al, 1996; Garufi et al, 1995; André et al, 1999). This difference in ORR is very possibly due to both the lack of eligibility criteria and the lack of enforced regular evaluations in the compassionate-use programs, which contrast with the uniform evaluation methodology required in clinical trials, and so influences the interpretation of results (Warr et al, 1984). Other factors resulting in the different ORRs may well be the lack of restriction

© 2001 Cancer Research Campaign on PS and the number of prior lines of chemotherapy for patients included in the present analysis. Another consideration is the importance of disease stabilization as a positive therapeutic outcome in the treatment of 5-FU-resistant advanced CRC patients (38.5% in this analysis) that is not considered in the determination of the ORR. This could explain why the TTP and OS are very close to those found in other studies, while the ORR is lower. The key parameter, OS, falls well within other published data from formal trials, and lends particular strength to the current analysis; as a matter of fact, patients potentially eligible for Phase II second-line CRC trials (good PS, ≤ 2 lines, good haemoglobin, etc.), show time-related progression parameters strictly similar to those reported in other studies (De Gramont et al, 1997; Gerard et al, 1998; André et al, 1999).

The present analysis confirms the activity of the oxaliplatin/5-FU ± FA combination as an active treatment for progressive 5-FU-resistant CRC patients. Although not strictly comparable, the results obtained in this heterogeneous, non-restricted cohort of 5-FU-resistant CRC patients, especially with respect to time-related parameters, compare favourably to those published for single-agent irinotecan following formal phase II–III trials including patients with similar disease characteristics (Rougier et al., 1998). The formal comparison between irinotecan and oxaliplatin/5-FU in 5-FU-pretreated CRC patients is warranted, but may come to be of secondary importance for therapeutic decision-making in the 5-FU refractory context in light of several reports showing their combination to be feasible and active in this patient population (Wasserman et al, 1999; Goldwasser et al, 2000; Bécouarn et al, 2001).

The patient's prognosis in such cases depends significantly on several factors discussed in this paper. The interest of oxaliplatin/5-FU as a therapeutic option in 5-FU-resistant cancer appears well supported by the results of the analysis of this 'real-life' prescription context. Its value appears enhanced when patients are treated earlier in their disease development and before the general deterioration of the patient's condition.

To conclude, this experience confirms the preclinical and clinical data reported in phase II–III studies concerning the synergy and activity of oxaliplatin in combination with 5-FU ± FA in 5-FU-resistant patients. Our analysis fails to show any strong relationship between 5-FU ± FA delivery modalities and time-related parameters. Finally, the efficacy of oxaliplatin-based combination treatment in this population is dependent on well-recognized factors related to patients' general status and pre-treatment history as well as the extent of their disease.

### ACKNOWLEDGEMENTS

This work was sponsored in part by grants from Debiopharm (Lausanne, Switzerland) and Sanofi-Synthelabo (Paris, France). We would like to thank Dr Bruno Lhote for his support of this project, and Mariko Johnson for her help in preparing the manuscript.

### REFERENCES

Advanced Colorectal Cancer Meta-Analysis Project (1992) Modulation of fluorouracil by leucovorin in patients with advanced colorectal cancer: evidence in terms of response rate. *J Clin Oncol* **10**: 896–903

André T, Bensmaine A, Louvet C, Francois E, Lucas V, Desseigne F, Beerblock K, Bouché O, Carola E, Merrouche Y, Morvan F, Dupont-André G and De Gramont A (1999) Multicenter Phase II Study of bimonthly High-dose Leucovorin, 5-Fluorouracil Infusion and Oxaliplatin for Metastatic Colorectal cancer resistant to the same leucovorin regimen (FOLFOX 3/4). *J Clin Oncol* **17**: 3560–3568

Bécouarn Y, Ychou M, Ducreux M, Borel C, Bertheault-Cvitkovic F, Seitz J, Nasca S, Nguyen T, Paillot B, Raoul J, Duffour J, Fandi A, Dupont-André G and Rougier P (1998) Phase II Trial of Oxaliplatin as First Line Chemotherapy in Metastatic Colorectal Cancer Patients. *J Clin Oncol* **16**: 2739–2744

Bécouarn Y, Gamelin E, Coudert B, Négrier S, Pierga JY, Raoul JL, Provencal J, Rixe O, Krisch C, Germau C, Bekradda M, Mignard D and Mousseau M (2001) A randomised multicenter phase II study comparing a combination of 5-fluorouracil (5-FU)/folinic acid (LV5-$FV_2$) and alternating irinotecan and oxaliplatin, with oxaliplatin/irinotecan in 5-FU-pretreated metastatic colorectal cancer patients *J Clin Oncol* (in press)

Behrens BC, Sickle-Santanello B and Martin E (1989) Protracted venous 5–Fluorouracil infusion and weekly cisplatin in metastatic colorectal cancer. *Proc Am Soc Clin Oncol* **8**: 126

Bertheault-Cvitkovic F, Jami A, Itzhaki M, Depres-Brummer P, Brienza S, Adam R, Kunstlinger F, Bismuth H, Misset JL and Lévi F (1996) Biweekly intensified ambulatory chronomodulated chemotherapy with oxaliplatin, fluorouracil, and leucovorin in patients with metastatic colorectal cancer. *J Clin Oncol* **14**: 2950–2958

Boring CC, Squires TS and Tong T (1992) Cancer statistics, 1992. *CA Cancer J Clin* **42**: 19–38

Cox DR (1970) *The Analysis of binary data*. London, England

Cox DR (1972) Regression models and life-tables (with discussion). *J R Stat Soc* **34**: 187–220

De Gramont A, Vignoud J, Tournigand C, Louvet C, André T, Varette C, Raymond E, Moreau S, Le Bail N and Krulik M (1997) Oxaliplatin with high-dose leucovorin and 5-fluorouracil 48 hour continuous infusion in pretreated metastatic colorectal cancer. *Eur J Cancer* **33A**: 214–219

De Gramont A, Figer A, Seymour M, Homerin M, Hmissi A, Cassidy J, Boni C, Cortes-Funes H, Cervantes A, Freyer G, Papamichael D, Le Bail N, Louvet C, Hendler D, De Braud F, Wilson C, Morvan F and Bonetti A (2000) Leucovorin and Fluorouracil with or without Oxaliplatin as first-line treatment in advanced colorectal cancer. *J Clin Oncol* **18**: 2938–2947

Douillard JY, Cunningham D, Roth A, Navarro M, James R, Karasek P, Jandik P, Iveson T, Carmichael J, Alakl M, Gruia G, Awad L and Rougier P (2000) Irinotecan combined with fluorouracil compared with fluorouracil alone as first-line treatment for metastatic colorectal cancer: a multicentre randomised trial *Lancet* **355**: 1041–1047

Falcone A, Cianci C, Pfanner E, Bertuccelli I, Muttini MP, Dargenio F, Ricci S and Conte PF (1994) Continuous-infusion 5-Fluorouracil in metastatic colorectal cancer patients pretreated with bolus 5-fluorouracil: clinical evidence of incomplete cross-resistance. *Ann Oncol* **5**: 291

Garufi C, Brienza S, Bensmaine A, Misset JL, Aschelter A, Pace R, Bertheault-Cvitkovic F, Terzoli E and Lévi F (1995) Addition of oxaliplatin (L-OHP®) to chronomodulated (CM) 5-fluorouracil (5-FU) and folinic acid (FA) for reversal of acquired chemoresistance in patients with advanced colorectal cancer (ACC). *Proc Am Soc Clin Oncol* **14**: 192

Gerard B, Bleiberg H, Michel J, Finet C, Recloux P, Diana D, Dufrane JP, Vandaele D, Hendlisz A, Confente C, Malarme M, Michaux D and Brienza S (1997) Oxaliplatin combined to 5-FU and Folinic Acid (5-FU/FA) as second or third line treatment in patients with advanced colorectal cancer (CRC). *Proc Am Soc Clin Oncol* **16**: 288a

Gerard B, Bleiberg H, Vandaele D, Gil T, Hendlisz A, Di Leo A, Fernez B and Brienza S (1998) Oxaliplatin combined to 5-fluorouracil and folinic acid: an effective salvage therapy in patients with advanced colorectal cancer. *Anti-Cancer Drugs* **9**: 301–305

Giacchetti S, Perpoint B, Zidani R, Le Bail N, Faggiuolo R, Focan C, Chollet P, Llory JF, Letourneau Y, Coudert B, Bertheault-Cvitkovic F, Larregain-Fournier D, Le Rol A, Walter S, Adam R, Misset JL and Lévi F (2000) Phase III multicenter randomized trial of Oxaliplatin added to chronomodulated Fluorouracil-Leucovorin as first-line treatment of Metastatic Colorectal Cancer. *J Clin Oncol* **18**: 136–147

Goldwasser F, Gross-Goupil M, Tigaud JM, Di Palma M, Marceau-Suissa J, Wasserman E, Yovine A, Misset JL and Cvitkovic E (2000) Dose escalation of CPT-11 in combination with oxaliplatin using an every two weeks schedule: A phase I study in advanced gastrointestinal cancer patients. *Ann Oncol* **11**: 1463–1470

Graf W, Pahlman L, Bergström R and Glimelius B (1994) The relationship between an objective response to chemotherapy and survival in advanced colorectal cancer. *Br J Cancer* **70**: 559–563

Hill M, Norman A, Cunningham D, Findlay M, Nicolson V, Hill A, Iveson A, Evans C, Joffe J and Nicolson M (1995) Royal Mardsen Phase III Trial of

© 2001 Cancer Research Campaign

Fluorouracil With or Without Interferon Alfa-2b in Advanced Colorectal Cancer. *J Clin Oncol* **13**: 1297–1302

Iyer PR, Moran EM, Kaneshiro CA, Ottenheimer EJ, El Sayad NI, Blitzer JB and Charter RA (1988) Chemotherapy with Cisplatinum (CDDP) and 5FU in Metastatic Colorectal Cancer. *Proc Am Soc Clin Oncol* **7**: 114

Izzo J, Fandi A, Villalobos W, Azli N, Bachouchi M, Levin FM, Benahmed M, Armand J, Rougier P and Cvitkovic E (1994) Low-Dose Protracted Continuous 5-Fluorouracil Infusion in Solid Tumors. *Journal of Infusional Chemotherapy* **4**: 135–139

Lévi F, Misset JL, Brienza S, Adam R, Metzger G, Itzhaki M, Caussanel JP, Kunstlinger F, Lecouturier S, Descorps-Declere A, Jasmin C, Bismuth H and Reinberg A (1992) A chronopharmacologic phase II clinical trial with 5-fluorouracil, folinic acid, and oxaliplatin using an ambulatory multichannel programmable pump. *Cancer* **69**: 893–900

Lévi F, Perpoint B, Garufi C, Focan C, Chollet P, Depres-Brummer P, Zidani R, Brienza S, Itzhaki M, Iacobelli S, Kunstlinger F, Gastiaburu C and Misset JL (1993) Oxaliplatin activity against metastatic colorectal cancer: a phase II study of 5-day continuous venous infusion at circadian-rhythm modulated rate. *Eur J Cancer* **29A**: 1280–1284

LoRusso P, Pazdur R and Redman SG (1989) Low-dose continuous infusion 5-fluorouracil and cisplatin: Phase II evaluation in advanced colorectal carcinoma. *J Clin Oncol* **12**: 486–490

Machover D, Diaz-Rubio E, De Gramont A, Schilf A, Gastiaburu C, Brienza S, Itzhaki M, Metzger G, N'Daw D, Vignoud J, Abad A, Francois E, Gamelin E, Marty M, Sastre J, Seitz J and Ychou M (1996) Two consecutive phase II studies of oxaliplatin (L-OHP) for treatment of patients with advanced colorectal carcinoma who were resistant to previous treatment with fluoropyrimidines. *Ann Oncol* **7**: 95–98

Meyer V, Delva R, Gamelin E, Lamezec B, Maillart P, Danquechin-Dorval E, Lortholary A, Boisdron-Celle M, Maigre M, Brienza S, Cvitkovic E and Larra F (1997) Oxaliplatin (LOHP) and 5-fluorouracil (5-FU) synergism in advanced colorectal cancer patients (ACRC). *Eur J Cancer* **33**: S167

Miller AB, Hoogstraten B, Staquet M and Winkler A (1981) Reporting results of cancer treatment. *Cancer* **47**: 207–214

Petrelli NJ, Madejewicz S and Rustum Y (1989) Combination chemotherapy of cisplatin and 5-fluorouracil for advanced colorectal adenocarcinoma. *Cancer Chemother Pharmacol* **23**: 57–60

Raymond E, Chaney S, Taamma A and Cvitkovic E (1998a) Oxaliplatin: a review of preclinical and clinical studies. *Ann Oncol* **9**: 1053–1071

Raymond E, Faivre S, Woynarowski J and Chaney S (1998b) Oxaliplatin: mechanism of action and antineoplastic activity. *Semin Oncol* **25**: 4–12

Rougier P, Van Cutsem E, Bajetta E, Niederle N, Possinger K, Labianca R, Navarro M, Morant R, Bleiberg H, Wils J, Awad L, Herait P and Jacques C (1998) Randomised trial of irinotecan versus fluorouracil by continuous infusion after fluorouracil failure in patients with metastatic colorectal cancer. *Lancet* **352**: 1407–1412

Saltz L, Cox JV, Blanke C, Rosen LS, Fehrenbacher L, Moore M, Maroun JA, Ackland S, Locker P, Pirotta N, Elfring G and Miller LL (2000) Irinotecan plus fluorouracil and leucovorin for metastatic colorectal cancer. Irinotecan Study Group. *N Engl J Med* **343**: 905–914

Streit M, Jaehde U, Stremetzne S, Ridwelski K, Kerz H, Strohbach F, Hohenberger P, Zwiebel FM, Hebart H, Bothig R, Kairies M, Zillig D, Schuchmann S, Warnecke S, Thiel E and Kreuser E (1997) Five-Day continuous infusion of 5-Fluorouracil and pulsed folinic acid in patients with metastatic colorectal carcinoma: An effective second-line regimen. *Ann Oncol* **8**: 1163–1165

Tournigand C, Louvet C, Quinaux E, Andre T, Lledo G, Flesch M, Ganem G, Landi B, Colin P, Denet C, Mery-Mignard D, Risse M-L, Buyse M and De Gramont A (2001) FOLFIRI followed by FOLFOX versus FOLFOX followed by FOLFIRI in Metastatic Colorectal Cancer (MCRC): Final results of a Phase III study. *Proc Am Soc Clin Oncol* **20**: 124a

Warr D, McKinney S and Tannock IF (1984) Influence of measurement error on assessment of response to anticancer chemotherapy: proposal for new criteria of tumor response. *J Clin Oncol* **2**: 1040

Wasserman E, Cuvier C, Lokiec F, Goldwasser F, Kalla S, Méry-Mignard D, Ould Kaci M, Bensmaine A, Dupont-André G, Mahjoubi M, Marty M, Misset JL and Cvitkovic E (1999) Combination of Oxaliplatin Plus Irinotecan in Patients With Gastrointestinal Tumors: Results of Two Independent Phase I Studies With Pharmacokinetics. *J Clin Oncol* **17**: 1751–1759

Whitehead RP, Fleming T, Macdonald JS, Ahmann FR, Garewal H and Kuebler J (1991) A Phase II study of 5-Fluorouracil plus cisplatin for metastatic colorectal adenocarcinoma: a south-west oncology group (SWOG) study. *Invest New Drugs* **9**: 345–347

© 2001 Cancer Research Campaign
